# Foundation Foods reveals significant changes in farmed Atlantic salmon relative to Standard Reference Legacy data

**DOI:** 10.3389/fnut.2026.1870100

**Published:** 2026-08-03

**Authors:** Michael R. Bukowski, Robert Goldschmidt, W. Craig Byrdwell, Kyle McKillop, Katherine M. Phillips, Pamela Pehrsson, Patrick Sullivan, Naomi Fukagawa

**Affiliations:** 1 USDA-Agricultural Research Service, Beltsville Human Nutrition Center, Beltsville, MD, United States; 2 Department of Biochemistry, Virginia Tech, Blacksburg, VA, United States

**Keywords:** farmed Atlantic salmon, FoodData Central, human nutrition, N3 fatty acids, tilapia

## Abstract

Food composition databases are critical for dietary surveillance and commonly used in epidemiological research but must be actively managed to reflect a dynamic food supply. This study presents a practical framework for flagging foods and nutrients where current individual-sample data may warrant review of legacy database values. Herein we compare new individual-sample data to legacy data obtained on composited samples. The fatty acid (FA) profile of farmed Atlantic salmon demonstrates fatty acid species differences not apparent at the class level. While total polyunsaturated FA (PUFA) appeared unchanged between the two datasets, the new profile was 2.4-fold higher in linoleic acid (from 0.900 to 2.152 g/100 g) and 3.4-fold higher in α-linolenic acid than legacy data (0.148 to 0.510 g/100 g), with decreases in eicosapentaenoic acid (EPA) and docosahexaenoic acid (DHA) concentrations. These nutritionally consequential shifts were flagged using a proposed practical concordance framework that compares current individual-sample Foundation Foods estimates with legacy SR values and prioritizes nutrients for further database review. This case study underscores the necessity of continuous investment in food analysis using modern, individual-sample methods. Implementing this framework may help the Foundation Foods sampling program prioritize foods and nutrients where current individual-sample data suggest meaningful divergence from legacy database values.

## 1 Introduction

In the United States of America, FoodData Central (FDC), the USDA's modern food composition database, underpins the nutrient estimates used in dietary guidance, food labeling, and the National Health and Nutrition Examination Survey (NHANES) (1). Changes within reported data have downstream consequences for public health assessment and dietary recommendations for the American population. The transition from the Standard Reference Legacy (SR) dataset that was last updated in 2018 to Foundation Foods (FF) represented a fundamental shift in how USDA characterizes the nutrient content of the American food supply, and the present study demonstrates the impact of that shift.

FDC contains five datasets: FF, SR, the Food and Nutrient Database for Dietary Studies (FNDDS), Branded Foods Products Database, and Experimental Foods (EF). Both FF and SR serve as source data for FNDDS, the application database used to determine the nutrient content of foods reported in What We Eat in America, the dietary intake component of NHANES (2). While SR and FF are related, they are not interchangeable. The nutrient data in SR represent values derived from the literature, calculated from related foods, or determined through chemical analysis of composited samples collected from locations across the United States (3–6). These composite-based values capture a mean for a given nutrient in a food within the American food system but, by design, cannot characterize the variability among individual samples of the food. Assessing variability across all the nutrients for all foods covered by SR would be cost prohibitive (1). FF addresses this limitation directly using a smaller number of samples. Nutrients in FF are analyzed in individual, non-composited samples specifically to determine the nutritional variability within a food. The two datasets are complementary when the food supply is stable, but with rapid changes in where/how food is produced, the older SR values may no longer accurately represent what consumers are eating. FF data then become essential for identifying and quantifying those changes, highlighting foods and nutrients which may merit a larger nation-wide study.

Seafood provides an ideal test case for evaluating the relationship between SR and FF because the system that brings fish to the American plate spans both aquaculture and wild-caught sources at population scale (7). The National Fisheries Institute's list of the ten most consumed seafoods in the United States, based on the 2022 NOAA Fisheries of the United States report, includes a mixture of farmed species (shrimp, tilapia, catfish) and wild-caught species (tuna, Alaska pollock, cod), with salmon consumed in both wild and farmed forms (8, 9). Developments in farmed salmon aquaculture over the past two decades have driven a substantial transition from marine fish oil toward terrestrial plant-based oils in fish feeds, with documented changes in the resulting fatty acid composition of the fish (10, 11). These aquaculture-driven changes create a natural experiment. For farmed species, we hypothesized that the FF values for fatty acid components would be different from SR legacy values consistent with changes in aquaculture practices. For wild-caught species, we hypothesized that the SR population means would fall within the biological variance determined by FF analysis, confirming the continued relevance of the legacy data.

Given the primacy of SR values in the determination of FNDDS, a critical tool for national dietary surveillance, the framework developed here using FF results as guidance, will aid in for prioritizing expanded studies for replacement SR values.

## 2 Materials and methods

### 2.1 Sample selection and study design

Seafood selections were limited to fish that could be commercially purchased as whole skinless raw filets. Candidate species were drawn from the National Fisheries Institute's list of most consumed seafood species in 2022, based upon the 2022 NOAA Fisheries of the United States report. Species with existing entries in SR were selected and included Nile tilapia (*Oreochromis niloticus*), Atlantic cod (*Gadus morhua*), farmed channel catfish (*Ictalurus punctatus*), wild-caught sockeye salmon (*Oncorhynchus nerka*), and farmed Atlantic salmon (*Salmo salar*). The SR comparison data were obtained from FoodData Central for tilapia (NDB 15261), cod (NDB 15015), farmed channel catfish (NDB 15234), sockeye salmon (NDB 15085), and farmed Atlantic salmon (NDB 15236), respectively.

The study was designed to test whether FF mean values for individual nutrients differed from corresponding SR values by more than a threshold of practical significance. Because SR provides no estimate of variance (a consequence of the composite sampling design), we initially used ±20% of the SR value as the boundary for meaningful difference. This threshold draws on the compliance framework established under 21 CFR 101.9(g), which defines tolerance limits for nutrient content relative to declared label values (12). It is adapted here as a practical benchmark for food composition comparisons in the absence of historical variance data. The null hypothesis is that the FF mean for a given nutrient falls within ±20% of the corresponding SR value (Equation 1). A target sample size of *n* = 8 per fish species was determined by power analysis for a one-sample *t*-test with α = 0.05 (two-sided) and 80% statistical power, assuming a standardized effect size of d = 1.0 (13).


$$\begin{array}{l} Null\;hypotheseis:\;80\%<\;\frac{N_{FF}}{N_{SR}}\times 100\;<120\% \end{array}$$ (1)

Here, the ±20% threshold was used as a practical threshold for potentially meaningful divergence from SR values. Because SR Legacy values generally do not provide sample-level variance, the FF mean ± 2 SD was used as a descriptive screening criterion to assess whether SR values fell within the observed variability of current FF samples. This 2 SD screen was not intended as a formal equivalence test or as evidence that two values derive from the same underlying population. Additionally, one sample of Atlantic cod (FDC sample ID 2684515) the chain of custody for the sample submitted for FAMES analysis was disrupted. While this did not impact proximate analysis, for fatty acid composition of Atlantic cod n=7.

### 2.2 Sample preprocessing

Raw seafood samples were collected regionally in the national capital region (Maryland and District of Columbia) and western Virginia from retail outlets throughout September and October 2023. Eight convenience samples from each fish classification, weighing approximately 1 kg per fish type per retail outlet, were collected. Samples were immediately frozen and homogenized in liquid nitrogen using a 6-L Robot Coupe Blixer food processor (Robot Coupe USA, Jackson, MS) until a fine powder was obtained. Portions of each powdered fish sample were stored in sealed glass jars under residual nitrogen at −60 °C until analyzed.

### 2.3 Proximate analysis

Moisture was determined by vacuum drying following a modified AOAC 950.46 method (14). Analysis of proximate components was performed by a commercial analytical laboratory following methods AOAC 950.54 for total fat, AOAC 991.20 (Kjeldahl) for protein, and AOAC 945.46 for ash (14).

### 2.4 Lipid extraction and fatty acid methyl ester preparation

For FA analysis, samples were thawed at 4 °C and 0.17 g to 2.4 g portions were weighed into 50 mL glass screw-top tubes with Teflon-lined caps and extracted using a modified Folch extraction (15). To each sample, 20 mL of 2:1 chloroform: methanol and 1 mL of internal standard solution (0.5066 mg/mL methyl heneicosanoate, FA 21:0, in heptane) were added. Samples were magnetically stirred, with periodic vortex mixing, for 60 min then transferred to 125 mL separatory funnels. Tubes were rinsed with a second 10 mL of 2:1 chloroform: methanol, which was combined in the separatory funnels. After vigorous shaking, 7 mL of aqueous 0.9% KCl were added (16). Solutions were vigorously shaken and left to separate overnight at 4 °C. The lower organic layer was collected and the solvent removed under a stream of nitrogen gas at ambient temperature.

Residues were dissolved in 1.5 mL isooctane and combined with 0.25 mL of 2 M potassium hydroxide in methanol. After 30 s of vortex mixing, samples were left to stand at room temperature for 30 min. The isooctane layer was transferred to 2 mL vials for analysis by gas chromatography with flame ionization detection (GC-FID) and gas chromatography-mass spectrometry with chemical ionization (CI-GC-MS). Samples were processed and run at random in batches of 7–8 with a quality control sample of canned salmon (*Oncorhynchus gorbuscha*).

### 2.5 GC-FID and GC-CI-MS analysis

Fatty acid methyl esters were analyzed by GC-FID on an Agilent 6890N gas chromatograph (Agilent Technologies, Santa Clara, CA, USA) with a split/splitless injector (250 °C, 50:1 split). Separation was performed on a Supelco SP-2560 column (100 m × 0.25 mm, 0.2 μm film thickness, Supelco, Bellefonte, PA, USA) with a 1.0 mL/min hydrogen carrier gas flow. The oven profile was as follows: initial temperature was 70 °C, increased by 4 °C/min to 140 °C, increased by 2 °C/min to 160 °C, then by 1 °C/min to 200 °C, then by 2 °C/min to 220 °C with a 15 min hold. The total run time was 92.5 min.

Identity confirmation was performed by CI-GC-MS using a 5975C/7890A GC-MSD-FID (Agilent Technologies, Santa Clara, CA, USA) with a split/splitless injector (250 °C, 40:1 split). Separation was performed on a Supelco SP-2560 column (100 m × 0.25 mm, 0.2 μm film thickness, Supelco, Bellefonte, PA, USA) with a 1.0 mL/min helium carrier gas flow. The oven profile was as follows: initial temperature was 70 °C, increased by 2 °C/min to 140 °C, then by 1 °C/min to 180 °C, then by 2 °C/min to 240 °C with a 15 min hold. The total run time was 120 min. The mass spectrometer was operated in positive chemical ionization mode with methane reagent gas, with scans from m/z 80–450 Daltons.

### 2.6 Fatty acid identification and quantitation

The carbon number and desaturation level for each species was determined by CI-GC-MS using the precursor ion mass for the [M]^+^, [M+H]^+^, or [M+NH4]^+^ ions. Full structural annotation was determined by retention time comparison with genuine standards and materials. These included multi-component FAME standards GLC 68B, GLC 463, methyl heneicosanoate (FA 21:0), and methyl tricosanoate (FA 23:0) from Nu-Check Prep (Elysian, MN, USA), methyl pentacosanoate from Santa Cruz Biotechnology (Dallas, TX, USA), and methyl hexacosanoate from Matreya LLC (Pleasant Gap, PA, USA). Eight geometric isomers of 9,12,15–18:3 were assigned based upon Standard L6031 (Sigma-Aldrich, St. Louis, MO, USA). Geometric isomers of 9,12–18:2 were assigned based upon an isomerized corn oil sample, previously described (17). Branched chain fatty acids were assigned based upon a bovine milk sample previously analyzed (18, 19). Fatty acids were named following the Lipid MAPS hierarchical nomenclature for fatty acyl moieties (Supplementary Table S1) (20, 21). Where relevant, common names were included in annotation.

Fatty acids were quantitated by GC-FID using relative response factors based upon individual fatty acid peak area ratio with the internal standard peak area (FA 21:0). Response factors for individual fatty acids were determined using commercial FAME standards (GLC 68B, GLC 463) with fatty acid concentrations ranging from 0.2 mg/mL to 2 mg/mL with a 0.5 mg/mL (FA 21:0) internal standard. These commercial standards contained the common SFA, MUFA and PUFA species found in the samples analyzed. Check standards of GLC 68B and GLC 463 drawn from the calibration set were run with each sample batch to confirm retention time and response factor stability. Peaks below 3.3-times signal to noise ratio were considered below the limit of detection and peaks below 10-times signal to noise were considered below the limit of quantitation. These were excluded from later calculations. FoodData Central sample IDs, metadata, and composition data are presented in Supplementary Table S2, and are also available at FoodData Central (https://fdc.nal.usda.gov/).

### 2.7 Quality control

An in-house matrix-matched quality control material consisting of canned red sockeye salmon (“Salmon CC”) was used to assess analytical variability. These control materials were cross-validated with standard reference materials to establish expected concentrations and maintained under carefully controlled conditions, specifically moisture-tight glass jars sealed under nitrogen and stored in darkness at −60 °C (22). Method performance was also evaluated using NIST Standard Reference Material 3275, Omega-3 and Omega-6 Fatty Acids in Fish Oil (National Institute of Standards and Technology, Gaithersburg, MD, USA). The reproducibility of the Salmon CC material across independent laboratories, including historical data from the National Food and Nutrient Analysis Program, was used in the assessment of data quality. Additionally blind duplicates of a fish sample within each class were performed.

### 2.8 Statistical analysis and chemometrics

Statistical analysis and plotting were performed in Igor Pro 8 (Wavemetrics, Inc., Portland, OR, USA). Additional analysis was performed in RStudio 2024.12.0 Build 467 (Posit Software, Boston, MA, USA) running R version 4.4.1 (51). Principal component analysis (PCA) analysis was performed using MetaboAnalyst 6.0 (52) with data normalized to the mass percent of each fatty acid in the samples and subsequent Pareto scaling. PCA based on fatty-acid mass-percent data was interpreted as exploratory visualization of broad fatty-acid profile patterns; database-update conclusions were based primarily on nutrient-level comparisons rather than PCA alone. Data were analyzed by one-way ANOVA with α of *P* < 0.05 and a 0.05 false discovery rate. Tukey's HSD was employed as a *post-hoc* test for significant interactions between groups.

## 3 Results

### 3.1 Proximate composition: SR and FF are concordant

The protein content values in FF were not significantly different from those reported in SR (Table 1). For total lipids, sockeye salmon, farmed tilapia, and farmed catfish had greater lipid content in FF than reported in SR, but SR values (representing composite samples) fell within two standard deviations (σ) of the FF means.

**Table 1:** Comparison of total fat (g/100 g) by Folch total lipid extraction as part of extraction for fatty acid analysis to crude fat determined by AOAC 950.54 Standard Reference (SR) and Foundation Foods (FF) total fat values in seafood samples expressed in grams /100 grams uncooked filet.

Sample		Protein (g/100 g)	Crude fat (CF) AOAC 950.54	Total lipid (TL), Folch	Δ (TL-CF)	*p*	∑FA	Δ Lipid (CF- ∑FA)	*p*
			Lipid extract (g/100 g)				Fatty Acid Tabulation (g/100 g)		
Atlantic salmon, farmed	SR	20.4	13.4	–	–	–	10.71	2.69	–
	FF	20.3 (0.83)	13.1 (1.89)	17.83 (4.11)	4.72	0.0066	12.14 (1.24)	0.97	0.260
Sockeye Salmon, wild	SR	22.2	4.69	–	–	–	3.323	1.37	–
	FF	22.3 (0.62)	4.94 (0.94)	7.35 (2.10)	2.42	0.0062	3.65 (0.66)	1.29	0.014
Tilapia, farmed	SR	20.1	1.70	–	–	–	1.446	0.25	–
	FF	19.0 (1.45)	2.49 (0.76)	2.37 (0.59)	−0.12	0.726	1.98 (0.67)	0.50	0.065
Atlantic cod, wild	SR	17.8	0.67	–	–	–	0.456	0.21	–
	FF	16.1 (3.03)	0.67 (0.16)	0.55 (0.08)	−0.12	0.098	0.157 (0.062)	0.51	1.4 x 10^−5^
Channel catfish, farmed	SR	15.2	5.94	–	–	–	5.052	0.89	–
	FF	16.5 (0.88)	7.31 (1.23)	9.49 (2.71)	2.18	0.040	6.36 (1.32)	0.94	0.103

Values in parentheses represent standard deviation. Values for p represent the significance of the difference between AOAC 950.54 compared to either Folch extraction and summation of all detected fatty acids as determined by a two-tailed Welch's T-test with unequal variance (*n* = 8 per group in FF, α = 0.05).

The Folch method, considered the gold standard for quantitative total lipid recovery, extracts both polar membrane lipids and nonpolar lipids such as triacylglycerols (23). Acid hydrolysis followed by petroleum ether extraction, as used in AOAC 950.54 for proximate fat determination, yields the mass of fatty acids from both neutral and polar lipid fractions, while ether extraction alone recovers primarily nonpolar lipids (24). In Table 1, lipid recovery from the Folch method (both as gravimetric total lipid and as the sum of fatty acids) was compared to AOAC 950.54 from proximate analysis. Farmed Atlantic salmon, wild sockeye salmon, and channel catfish had significantly higher lipid recovery (*p* < 0.05) under the Folch method than leaner fish such as tilapia and cod, for which the two methods yielded values that were not significantly different.

For all five species, the proximate composition data establish concordance between SR and FF. The macronutrient profile of these fish has not changed in ways that exceed the expected biological variability over the nearly 2 decades of SR data.

### 3.2 Fatty acid detection, annotation, and quality control

**Figure 1:**
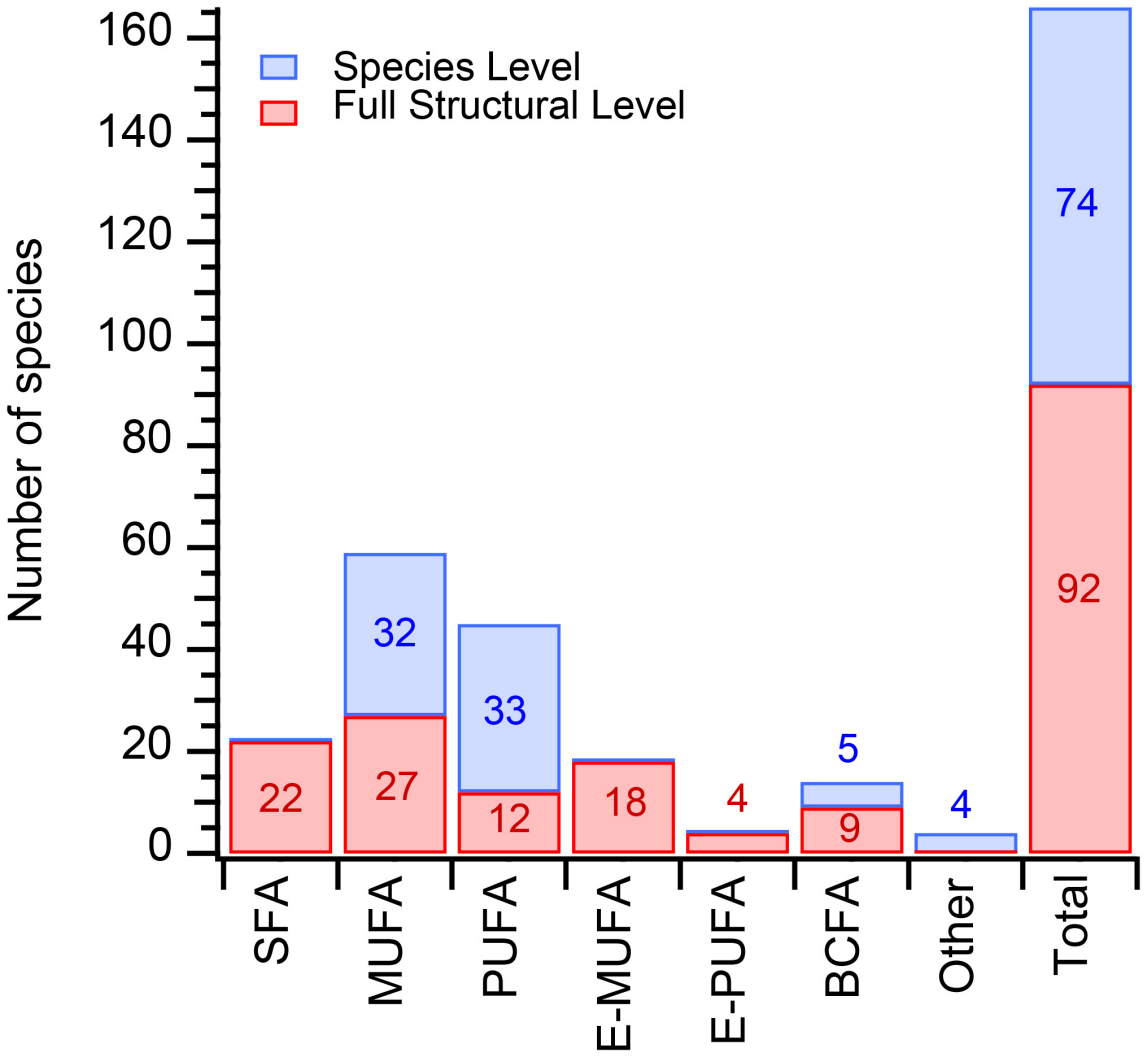
Tabulation of detected fatty acid species with level of characterization. Full structural characterization allows for assignment of a common name based upon comparison with a pure compound.

Using the Lipid MAPS hierarchical nomenclature (21), 166 fatty acid residues were identified across all fish species. These included 40 saturated, 77 monounsaturated, and 49 polyunsaturated species (Figure 1). Structural annotation was performed by CI-GC-MS using the precursor ion mass for the [M]^+^, [M+H]^+^, or [M+NH_4_]^+^ ions, with full structural assignment confirmed by retention time alignment with purified standards where available. For monounsaturated species, published elution order data were used to infer structural annotation in the absence of purified standards (19). Through this combined approach, 92 fatty acids were identified at full structural resolution. These comprised 22 SFA, 27 MUFA, 12 PUFA, 18 E-MUFA, 4 E-PUFA, and 9 BCFA. This represents a substantially more detailed fatty acid characterization than is currently available in the SR database (Supplementary Table S1).

The sum of all fatty acids at both annotation levels is shown in Table 1, compared to total lipid by AOAC. While summed fatty acid values were lower in all cases than AOAC 989.05 determinations, the difference was significant (*p* < 0.05) only for wild-caught species (sockeye salmon and cod), where inter-sample variability was low.

### 3.3 Fatty acid class totals: apparent concordance masks species-level divergence

**Figure 2:**
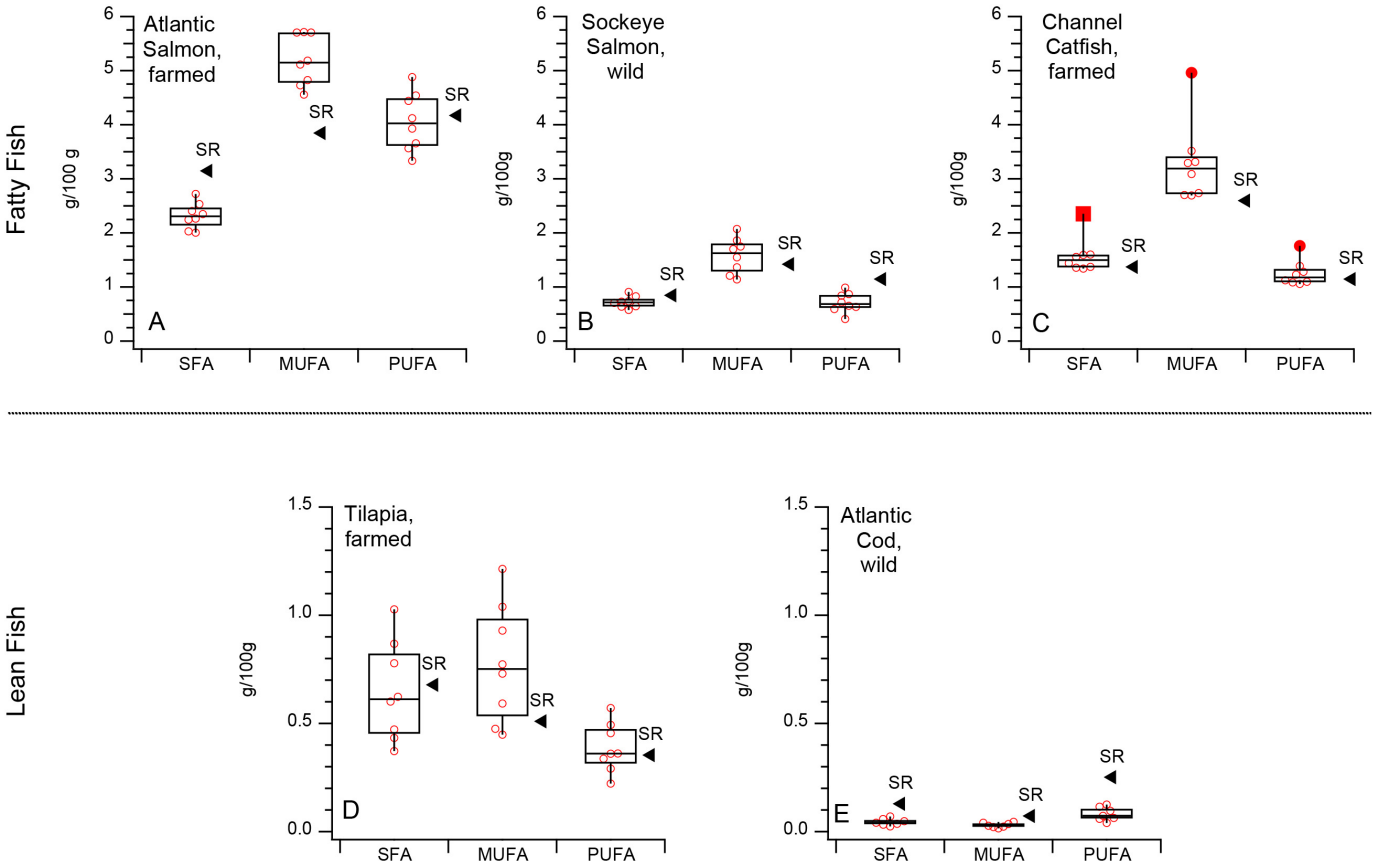
Box plots displaying variability in total saturated fatty acid (SFA), monounsaturated fatty acid (MUFA), and polyunsaturated fatty acid (PUFA) content for **(A)** farm-raised Atlantic salmon (*n* = 8), **(B)** wild-caught sockeye salmon (*n* = 8), **(C)** farm-raised catfish, **(D)** farm-raised tilapia (*n* = 8), and **(E)** wild-caught cod (*n* = 7). Quartiles determined by Tukey's method, with whiskers representing the minimum and maximum measures. Outlier values (1.5 IQR) and far outlier values (3 IQR) are indicated as filled circles and filled boxes, respectively. Values reported in SR are shown on the graph with an arrow. The SR value for cod PUFA content exceeds the scale and is indicated in parentheses.

The totals for SFA, MUFA, and PUFA content were calculated using only fatty acid species with full structural identification and compared with published SR values (Table 2, Figure 2). At this level of aggregation, the farmed species appeared largely concordant between SR and FF. For farmed Atlantic salmon, SFA content was 0.73 g/100 g lower and MUFA content was 1.30 g/100 g higher than SR values, while total PUFA content appeared unchanged (Figure 2A). For farmed channel catfish and tilapia, SR values for all three classes fell within the FF sampling variability (Figures 2C, D). The SR values for wild sockeye salmon were within the FF range for SFA and MUFA, although the historical SR PUFA value landed just above the maximum value measured in FF (Figure 2B). Cod showed uniformly lower values for SFA, MUFA, and PUFA than previously reported in SR. Including fatty acids at all annotation levels increased cod MUFA and PUFA values by 0.030 g/100 g and 0.079 g/100 g, respectively, but this was insufficient to account for the difference from historical SR values (Figure 2E).

**Table 2:** Comparison of saturated fatty acid (SFA), monounsaturated fatty acid (MUFA), and polyunsaturated fatty acid (PUFA) totals for Standard Reference (SR) and Foundation Foods (FF) seafood samples expressed in grams /100 grams uncooked filet.

Sample		SFA (g/100 g)	MUFA (g/100 g)	PUFA (g/100 g)
Atlantic salmon, farmed	SR	3.05	3.77	3.88
	FF	2.32 (0.37)	5.07 (0.807)	4.12 (0.37)
Sockeye Salmon, wild	SR	0.814	1.37	1.12
	FF	0.719 (0.108)	1.58 (0.326)	0.712 (0.181)
Tilapia, farmed	SR	0.585	0.498	0.363
	FF	0.669 (0.255)	0.800 (0.300)	0.393 (0.117)
Atlantic cod, wild	SR	0.131	0.094	0.231
	FF	0.042 (0.016)	0.029 (0.011)	0.078 (0.032)
Channel catfish, farmed	SR	1.31	2.57	1.12
	FF	1.58 (0.33)	3.34 (0.75)	1.24 (0.23)

Totals represent only species for which full structural assignment could be made based upon mass spectrometry and retention time comparison with a pure standard. Values in parentheses represent standard deviation (*n* = 8 per group in FF, for cod *n* = 7).

**Figure 3:**
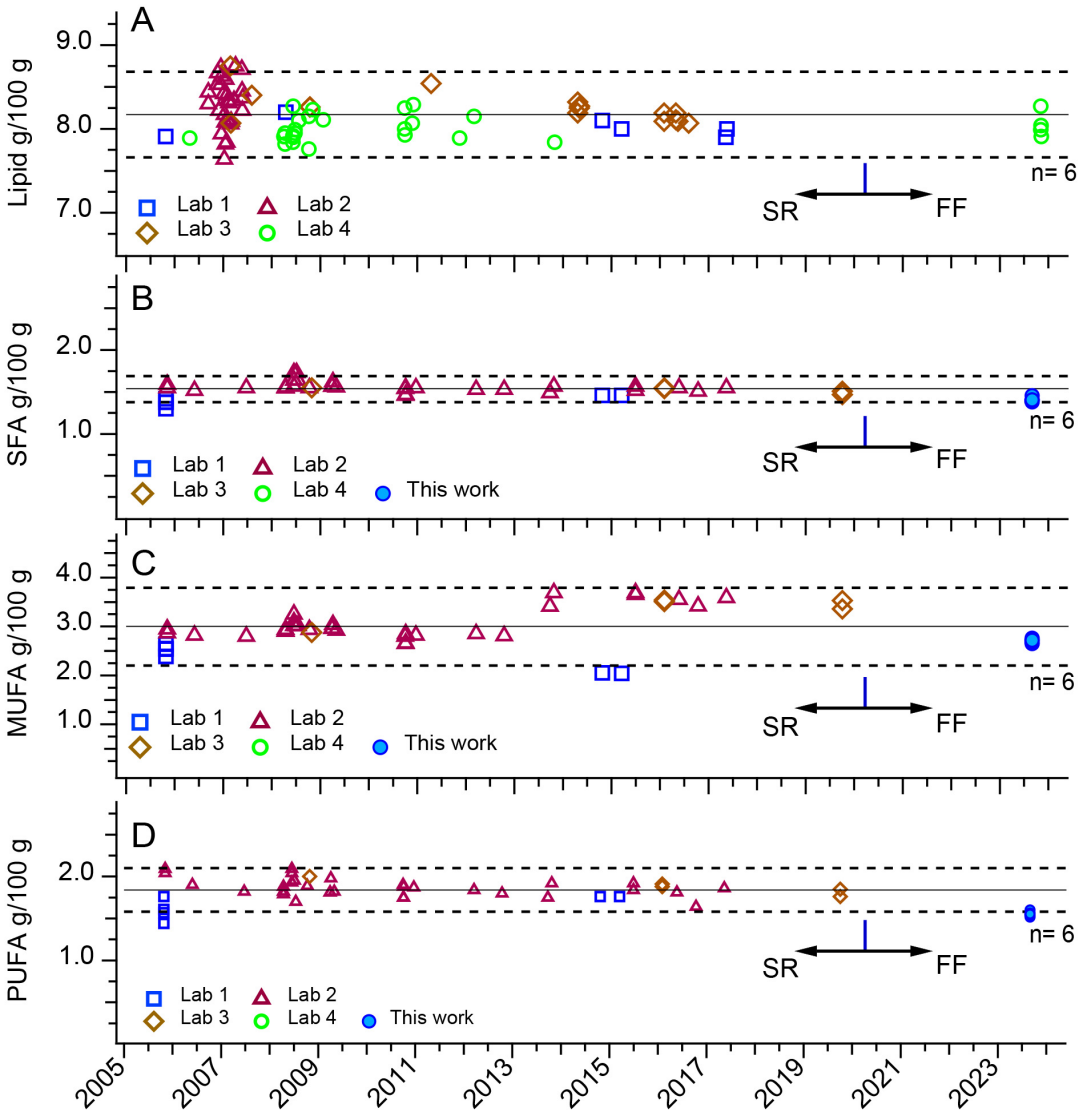
Reproducibility of salmon control material (“Salmon CC”) for **(A)** total fat, **(B)** total saturated fat (SFA), **(C)** total monounsaturated fatty acid (MUFA), and **(D)** total polyunsaturated fatty acid (PUFA). Samples were analyzed by four independent laboratories as part of the National Food and Nutrient Analysis Program for SR and later Foundation Foods. The solid line represents the mean value for all laboratories, with dashed lines representing two standard deviations above and below the mean. Work presented for FF comes from six technical replicates run contemporaneously with fish samples reported in this study.

The reproducibility of the control material support analytical stability, making analytical drift an unlikely explanation for the observed difference. Analysis of this material by four commercial labs over more than a decade of data acquisition for SR Legacy produced mean values for total lipid, SFA, MUFA and PUFA, along with variances for these quantities Figures 3A–D, respectively. The QC results for this study are within two standard deviations for all terms, indicating that the changes observed in the experimental samples were not the consequences of possible differences in methodology.

### 3.4 Individual fatty acid species

#### 3.4.1 Saturated fatty acids

SFA species are reported in Table 3. Fatty acids at or below 10 carbons were not observed above the limit of detection, and lauric acid (FA 12:0) was detected at or below 6 mg/100 g. Palmitic acid (FA 16:0) was the largest contributor to SFA content for all fish species. In farmed Atlantic salmon, lower palmitic and myristic acid (FA 14:0) content accounted for much of the difference in SFA concentration between SR and FF.

**Table 3:** Comparison of selected saturated fatty acid (SFA) for Standard Reference (SR) and Foundation Foods (FF) seafood samples.

Sample		FA 12:0	FA 14:0	FA 16:0	FA 18:0
		Lauric acid	Myristic acid	Palmitic acid	Stearic acid
Atlantic salmon, farmed	SR	0	0.556	1.88	0.495
	FF	0.006 (0.0007)	0.248 (0.037)	1.45 (0.14)	0.475 (0.057)
Sockeye Salmon, wild	SR	0.002	0.126	0.549	0.104
	FF	0.001 (0.0003)	0.119 (0.025)	0.483 (0.072)	0.088 (0.020)
Tilapia, farmed	SR	0	0.053	0.424	0.107
	FF	0.003 (0.0029)	0.051 (0.015)	0.475 (0.187)	0.123 (0.052)
Atlantic cod, wild	SR	0	0.009	0.091	0.03
	FF	< 0.0001	0.002 (0.001)	0.030 (0.013)	0.009 (0.002)
Channel catfish, farmed	SR	0.002	0.056	0.939	0.284
	FF	0.001 (0.0003)	0.069 (0.014)	1.138 (0.228)	0.334 (0.097)

Species presented had full structural assignment based upon mass spectrometry with retention time comparison to a pure standard and are presented with their common name. Quantities are expressed in grams /100 grams of uncooked filet. Values in parentheses represent standard deviation (*n* = 8 per group in FF, for cod *n* = 7).

#### 3.4.2 Oleic acid and MUFA

**Figure 4:**
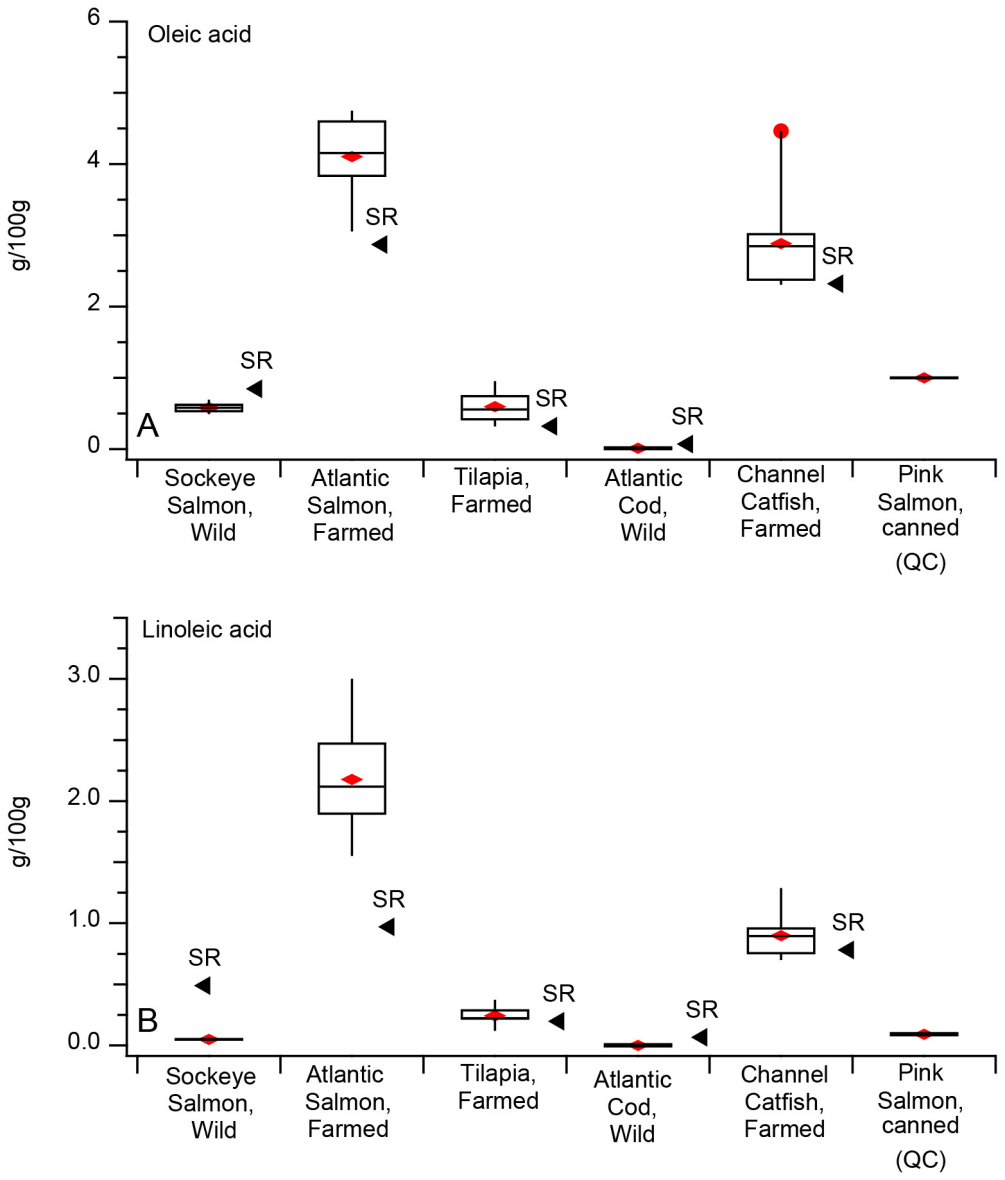
Box plots displaying variability in **(A)** oleic acid (FA 18:1, 9Z) and **(B)** linoleic acid (FA 18:2, 9Z, 12Z). Wild sockeye salmon, farm-raised Atlantic salmon, farm-raised tilapia, wild Atlantic cod, and farm-raised channel catfish (*n* = 8 per type, *n* = 7 for cod) are shown with canned salmon quality control (6 replicates) for analytical variability. Mean values shown as filled diamonds. Quartiles determined by Tukey's method, with whiskers representing minimum and maximum measures. Outlier values (1.5 IQR) and far outlier values (3 IQR) are indicated as filled circles and filled boxes, respectively. Values reported in SR are shown on the graph with an arrow.

Concurrent with the lower SFA content for farmed Atlantic salmon, the concentration of oleic acid (FA 18:1, 9Z) was 1.54 g/100 g higher than historic SR values (Table 4, Figure 4A). While the mean values for oleic acid in farmed tilapia and farmed catfish were also greater in FF relative to SR (by 0.228 g/100 g and 0.614 g/100 g, respectively), these differences fell within sampling variability (Figure 4A). The oleic acid content of wild sockeye salmon was lower in FF (0.582 g/100 g) than in SR (0.701 g/100 g).

**Table 4:** Comparison of selected monounsaturated fatty acids (MUFA) and polyunsaturated fatty acids (PUFA) for Standard Reference (SR) and Foundation Foods (FF) seafood samples.

Sample		FA 18:1(9Z)	FA 18:2	FA 18:3	FA 20:5	FA 22:5	FA 22:6	EPA + DHA	Total	Total
		Oleic acid	Linoleic acid (N6)	α-linolenic acid (N3)	EPA (N3)	DPA (N3)	DHA (N3)		N3^1^	N6^2^
Atlantic salmon, farmed	SR	2.72	0.900	0.148	0.862	0.393	1.100	1.962	2.50	1.07
	FF	4.26 (0.41)	2.152 (0.351)	0.510 (0.083)	0.312 (0.056)	0.163 (0.026)	0.577 (0.058)	0.889	1.78	2.31
Sockeye Salmon, wild	SR	0.701	0.151	0.047	0.251	0.079	0.471	0.722	0.848	0.154
	FF	0.582 (0.072)	0.049 (0.008)	0.020 (0.005)	0.210 (0.065)	0.068 (0.020)	0.324 (0.087)	0.534	0.732	0.085
Tilapia, farmed	SR	0.379	0.158	0.033	0.005	0.043	0.086	0.091	0.170	0.181
	FF	0.607 (0.240)	0.248 (0.073)	0.018 (0.006)	0.002 (0.0008)	0.007 (0.002)	0.029 (0.007)	0.031	0.119	0.297
Atlantic cod, wild	SR	0.061	0.005	0.001	0.064	0.01	0.12	0.184	0.196	0.027
	FF	0.011 (0.004)	0.00097 (0.00058)	2.3 x 10^−4^ (2.1 x 10^−4^)	0.0181 (0.0075)	0.0026 (0.0005)	0.0533 (0.0204)	0.071	0.075	0.0057
Channel catfish, farmed	SR	2.31	0.755	0.054	0.017	0.015	0.057	0.074	0.145	0.817
	FF	3.02 (0.63)	0.938 (0.175)	0.062 (0.010)	0.0138 (0.0101)	0.013 (0.005)	0.045 (0.018)	0.058	0.138	0.981

Species presented had full structural assignment based upon mass spectrometry with retention time comparison to a pure standard and are presented with their common name. N3 and N6 designations are included where relevant. Quantities are expressed in grams /100 grams of uncooked filet. Values in parentheses represent standard deviation (*n* = 8 per group, except for cod *n* = 7). For FF values, range and 95% confidence intervals are presented in Supplementary Table S3. ^1^ Total N3 content includes, but is not limited to, ALA, stearidonic acid, EPA, DPA, and DHA. ^2^Total N6 content includes, but is not limited to, linoleic acid, γ-linolenic acid, and arachidonic acid.

#### 3.4.3 Linoleic acid and N6 PUFA

The concentration of linoleic acid (FA 18:2, 9Z, 12Z) in farmed Atlantic salmon increased 2.4-fold, from 0.900 g/100 g in SR to 2.152 g/100 g in FF (Table 4, Figure 4B). While the other farmed fish also had higher linoleic acid content in FF, the SR values remained within the sampling variability of the FF data (Figure 4B). The concentration of linoleic acid in wild sockeye salmon decreased by a factor of three, from 0.151 g/100 g (SR) to 0.049 g/100 g (FF).

#### 3.4.4 N3 PUFA

**Figure 5:**
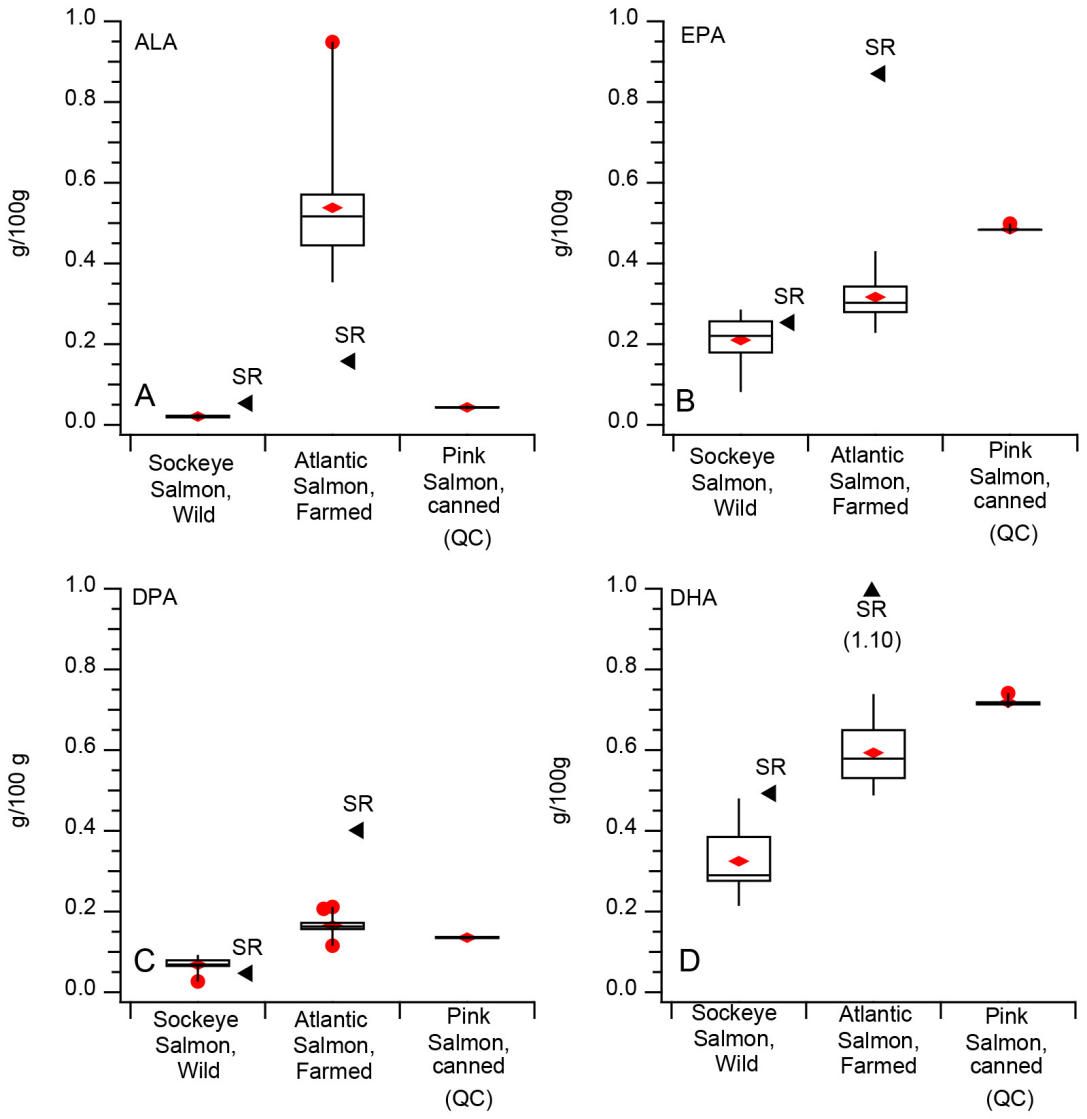
Box plots displaying variability in **(A)** α-linolenic acid (FA 18:3, 9Z, 12Z, 15Z), **(B)** eicosapentaenoic acid (FA 20:5, 5Z, 7Z, 11Z, 14Z, 17Z), **(C)** docosapentaenoic acid (FA 22:5, 7Z, 10Z, 13Z, 16Z, 19Z), and **(D)** docosahexaenoic acid (FA 22:6, 3Z, 6Z, 9Z, 12Z, 15Z, 18Z). Wild sockeye salmon, farm-raised Atlantic salmon (*n* = 8 per type), are shown with canned salmon quality control (6 replicates) for analytical variability. Mean values shown as filled diamonds. Quartiles determined by Tukey's method, with whiskers representing minimum and maximum measures. Outlier values (1.5 IQR) and far outlier values (3 IQR) are indicated as filled circles and filled boxes, respectively. Values reported in SR are shown on the graph with an arrow.

Four N3 PUFA species are reported for all samples (Table 4), with the most consequential changes occurring in farmed Atlantic salmon (Figure 5). The concentration of α-linolenic acid (ALA) increased from 0.148 g/100 g to 0.510 g/100 g, a 3.4-fold increase (Figure 5A). In contrast, the marine long-chain PUFA species decreased substantially. EPA decreased by 63.8%, DPA by 58.5%, and DHA by 47.5% (Figures 5B–D). As a result, the total N3 PUFA content of farmed Atlantic salmon decreased from 2.11 g/100 g to 1.78 g/100 g. The composition of that total has shifted dramatically, from predominantly marine-derived EPA and DHA toward plant-derived ALA, a change with direct implications for the nutritional value consumers can expect from this food. In wild sockeye salmon, EPA + DHA content decreased from 0.722 g/100 g to 0.534 g/100 g.

### 3.5 N6 fatty acid metabolism

**Figure 6:**
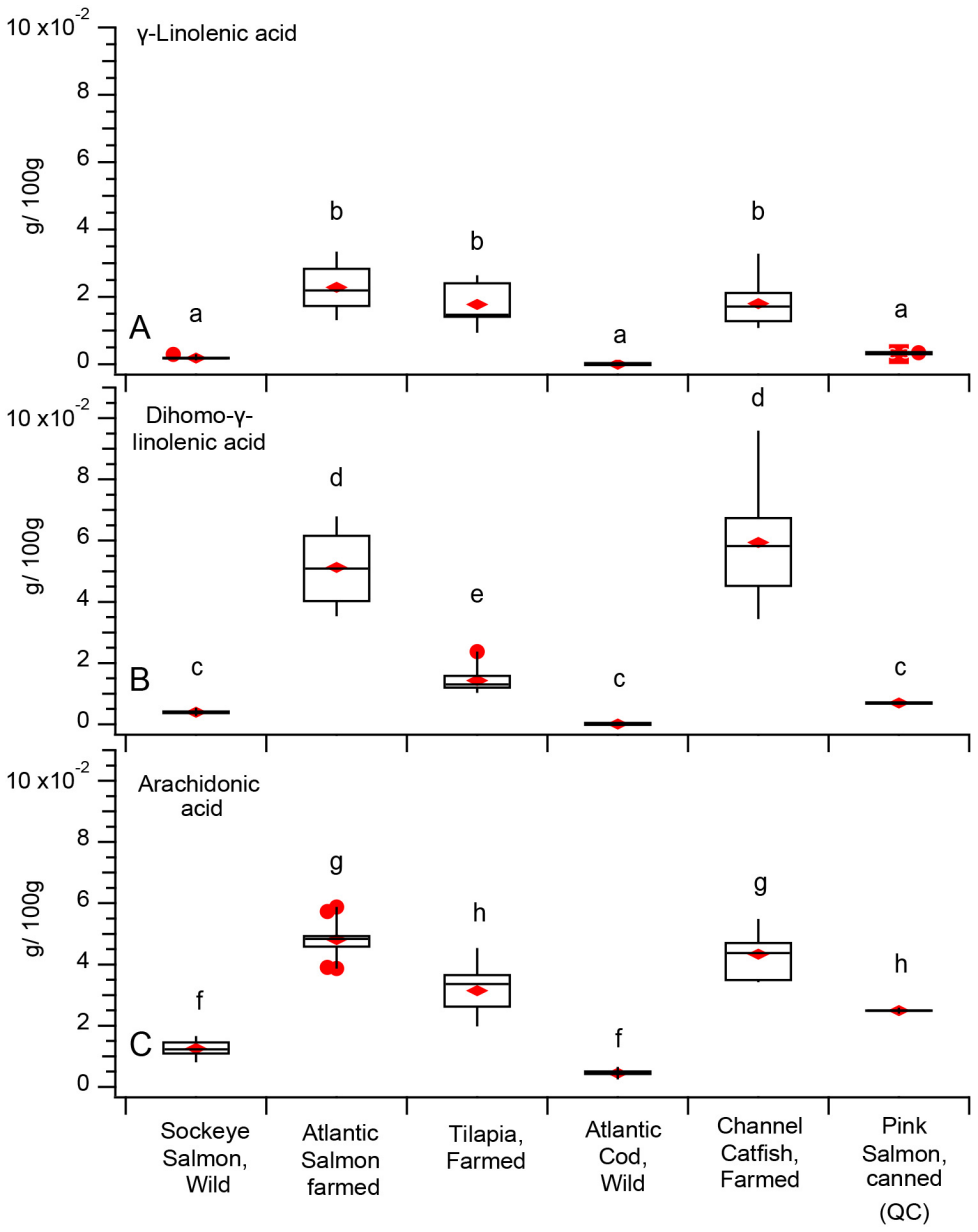
Box plots for downstream fatty acids in linoleic acid metabolism. **(A)** γ-linolenic acid (FA 18:3, 6Z, 9Z, 12Z), **(B)** dihomo-γ-linolenic acid (FA 20:3, 8Z, 11Z, 14Z), and **(C)** arachidonic acid (FA 20:4, 5Z, 8Z, 11Z, 14Z). Wild sockeye salmon, farm-raised Atlantic salmon (*n* = 8 per type), are shown with canned salmon quality control (6 replicates) for analytical variability. Mean values shown as filled diamonds. Quartiles determined by Tukey's method, with whiskers representing minimum and maximum measures. Outlier values (1.5 IQR) and far outlier values (3 IQR) are indicated as filled circles and filled boxes, respectively. *Post-hoc* relationships were tested by Tukey's HSD test (α = 0.05). Samples with common letters are not significantly different.

The downstream desaturation and elongation products of linoleic acid metabolism are shown in Figure 6. Within the FF dataset, farmed Atlantic salmon, farmed tilapia, and farmed channel catfish all had significantly higher levels of γ-linolenic acid (*p* < 0.05) relative to wild-caught species (Figure 6A), but the values among the farmed species were not significantly different from each other. For dihomo-γ-linolenic acid and arachidonic acid, the content in tilapia was lower than in the other farmed fish (*p* < 0.05) (Figures 6B, C).

### 3.6 Wild-caught and low-fat species: concordance with caveats

**Figure 7:**
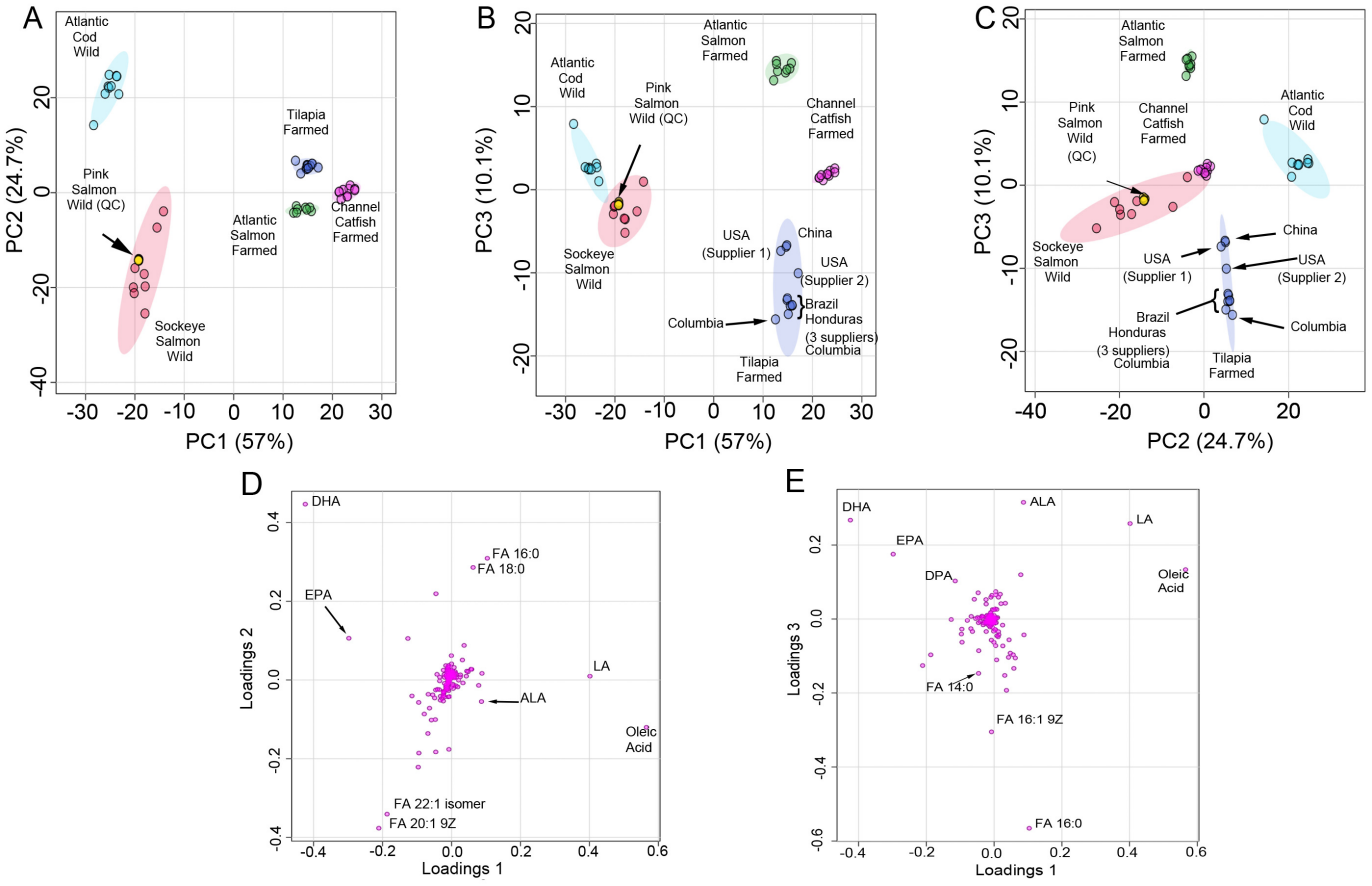
Principal component analysis (PCA) of fatty acid composition data normalized to total fatty acid content in each sample. Principal components (PC) are shown with the degree of variance explained by each component. **(A)** PC1 vs. PC2, **(B)** PC1 vs. PC3, **(C)** PC2 vs. PC3, **(D)** loading plots for PC1 and PC2, **(E)** loading plots for PC1 and PC3.

For wild sockeye salmon, the SR and FF values were largely concordant, with the exception of the PUFA total falling just outside the 2σ boundary and the notable decrease in linoleic acid noted in Section 3.4. The narrow biological variance observed in the canned salmon quality control material (Figures 7A–C) confirms that the broader distribution observed in wild fish reflects genuine population-level variability rather than analytical noise.

For farmed channel catfish and tilapia, SR values for fatty acid class totals fell within FF variability. For tilapia, however, the variance in fatty acid composition across individual samples exceeded 30%, substantially larger than the 20% threshold used in the study design. PCA analysis (Section 3.7) suggests that country of origin may account for much of this variation. Given the low overall fat content of tilapia (approximately 1.5 g/100 g), these compositional differences, while analytically real, may have limited nutritional relevance at typical consumption levels.

For cod, all fatty acid class totals were lower in FF than in SR. The low total fat content of cod (less than 1 g/100 g) means that the seasonal and biological variability inherent in a lean white fish translates to large relative differences in fatty acid concentrations. As with tilapia, these differences may have limited nutritional significance given the absolute fat content of the food.

### 3.7 Chemometric exploration

Chemometric analysis by PCA was performed on the same fatty-acid dataset used for univariate analyses to explore multivariate relationships that may impact future study design. When fatty acid data were normalized to total fatty acid content, PCA accounted for 91.9% of the variance with three components. Component 1 (57.0% of the variance) separated farmed from wild fish. Component 2 (24.7%) differentiated wild cod from wild salmon. Component 3 (10.1%) separated the three farmed species (Figures 7A–C).

The loading plots identify oleic acid, linoleic acid, and α-linolenic acid as the key drivers of the farmed-vs.-wild separation on PC1 (Figures 7D, E), precisely the fatty acids whose changes were documented in Section 3.4. The relative DHA content drove the separation on Component 2, distinguishing the low-fat Atlantic cod (where DHA constitutes a high proportion of total fatty acids) from the fattier wild sockeye salmon (Figures 7D, E). On Component 3, DHA content differentiated farmed Atlantic salmon from catfish and tilapia, while the relative palmitic and palmitoleic acid content further distinguished farmed tilapia from farmed channel catfish (Figure 7E).

Two additional features of the PCA warrant attention. The inclusion of six canned salmon quality control samples, which clustered tightly with wild sockeye salmon (Figures 7A–C), illustrates the narrow analytical variability achievable when a single material is analyzed repeatedly. And while the distribution of farmed Atlantic salmon and farmed channel catfish samples was considerably narrower than that of the wild-caught species, farmed tilapia showed a distinctive pattern. It was narrowly distributed on PC1 and PC2 but more widely separated on PC3, driven by palmitic and palmitoleic acid variation that the associated metadata suggests may reflect differences in country of origin (Figures 7D, E).

## 4 Discussion

### 4.1 A framework for evaluating FF and SR concordance

The SR Legacy dataset and the FF dataset were designed with fundamentally different objectives. SR was designed to capture population means for nutrients across the United States through composite sampling, a strategy that provided a single representative value for a food but necessarily sacrificed information about variability among individual samples. FF was developed to characterize the biological variability within the food supply by analyzing individual, non-composited samples, information that may impact precision nutrition as well as our understanding of the nutritional significance of variation in whole, minimally processed foods.

The question of when FF should replace rather than complement SR can be addressed through a straightforward criterion. When the SR value for a given nutrient falls within two standard deviations of the FF mean, the datasets may be considered broadly concordant for screening purposes. In this case, SR and FF are complementary. The former provides a historical population mean, and the latter characterizes the variability within that population. When the SR value falls outside this boundary, however, the two datasets are no longer describing the same food, and further investigation is warranted into the cause and nutritional significance of the difference.

This study demonstrates both conditions. For proximate composition across all five species, and for fatty acid class totals in most cases, SR and FF are concordant. The datasets are complementary with FF adding variability information that SR cannot provide. At the individual fatty acid level, however, a different picture emerges.

### 4.2 Farmed Atlantic salmon: aquaculture-driven change with nutritional consequences

The fatty-acid data for farmed Atlantic salmon represent the clearest case in this study where FF values suggest that legacy SR fatty-acid values may require review and potential updating, rather than being treated as directly interchangeable with current products. The increases in oleic acid, linoleic acid, and α-linolenic acid, concurrent with substantial decreases in EPA (63.8%), DPA (58.5%), and DHA (47.5%), are consistent with the well-documented transition in Atlantic salmon aquaculture from marine fish oil toward terrestrial plant-based oils in feed formulations (10, 11).

Like humans, salmon require LA and ALA as essential fatty acids, and like humans they have a low conversion rate of ALA to EPA and DHA (25, 26), therefore much of the EPA and DHA in salmon are a consequence of trophic concentration in of these fatty acids within the marine ecosystem (27). At the turn of the century it was recognized that increased farming of carnivorous fish like salmon would put unsustainable pressure on wild stocks of prey fish, impacting the commercial viability of aquaculture (28). Research into the terrestrial substitutes for fish oil included oils from soybean (29), rapeseed (30), sunflower (31), linseed (32), canola (33), camelina (34). Bell et al. demonstrated that the diminished DHA and EPA content in farmed salmon due to a terrestrial diet could be partially ameliorated by using fish oil in the finishing diet prior to harvest (35, 36). Consequently, even as the harvest of prey fish dropped 30% from 2000 to 2017, with fish oil prices more than doubling, the global output of fed fish tripled (37). Our study did not have access to feeds, production systems, supplier histories, and finishing diets, but with the decrease in EPA and DHA content with changing diet has been documented (10, 11, 38), this is a plausible explanation of the changes observed. Further, aquaculture researchers are incorporating oils from new transgenic plants capable of generating EPA and DHA, and microalgal oils rich in DHA, providing long chain N3 PUFA independent of marine fish inputs (33, 39, 40). These advances suggest we may see continued change in the EPA and DHA content of farmed salmon.

While the total N3 PUFA content of farmed Atlantic salmon decreased modestly (from 2.11 g/100 g to 1.78 g/100 g), the composition of that total has shifted substantially. It has moved from predominantly marine-derived EPA and DHA, which are the bioactive forms relevant to cardiovascular and neurological outcomes, toward plant-derived ALA, which requires metabolic conversion to EPA and DHA at rates that are limited in humans (41, 42). Any dietary intake estimate for EPA and DHA derived from the SR value for farmed Atlantic salmon could overstate the actual content of current market fish by more than 50%.

The situation with farmed Atlantic salmon is analogous, in principle, to the existing SR entry for wild Atlantic salmon (*Salmo salar*). Wild Atlantic salmon listed in SR is now a species protected under the United States Endangered Species act (43, 44), therefore the food described in the database is no longer common in the marketplace. In the case of farmed Atlantic salmon, the food still exists, but its fatty acid composition has changed to the point where the SR values no longer describe it accurately. In both cases, the SR data represent a historical artifact rather than a current nutritional reality.

### 4.3 Wild-caught and low-fat species. Biological variability vs. systematic change

For wild sockeye salmon, the general concordance between SR and FF supports the continued relevance of the legacy data, with the caveat that the decrease in linoleic acid content (from 0.151 g/100 g to 0.049 g/100 g) and the modest decrease in DHA content deserve further monitoring. These changes may reflect year-to-year variation in wild fish populations rather than a systematic shift in composition.

For farmed channel catfish, SR and FF values are concordant at both the class and individual fatty acid levels, suggesting that the current database values remain appropriate.

For tilapia, the greater-than-expected variance across individual samples (exceeding 30% for some fatty acid components) appears to be related to country of origin, as suggested by the PCA analysis. While this variance exceeds the 20% threshold applied in the study design, the low total fat content of tilapia (approximately 1.5 g/100 g) limits the nutritional impact of these compositional differences at typical consumption levels. The FF dataset adds value here by identifying a possible source of variability that the composite-based SR approach could not detect, information that may become more relevant as tilapia sourcing patterns continue to evolve. The exploratory visualization provided by PCA along with the country of origin metadata suggest important considerations for future study design. The possible country of origin effect does impact the other fish as catfish, sockeye salmon, and cod were sourced almost exclusively from USA producers, and the farmed salmon from Chilean producers (Supplementary Table S2). A larger study would be needed to assess the impact of regional feeding practices on the fatty acid composition of tilapia, and the viability of using fatty acid composition as a screen for country of origin.

For cod, the uniformly lower fatty acid values in FF relative to SR are likely attributable to seasonal variation (45–47). Cod is an extremely lean fish (less than 1 g total fat per 100 g), and small absolute changes in lipid content translate to large relative differences in fatty acid concentrations. As with tilapia, the low total fat content limits the nutritional significance of these differences.

### 4.4 Implications for FoodData Central and dietary surveillance

**Figure 8:**
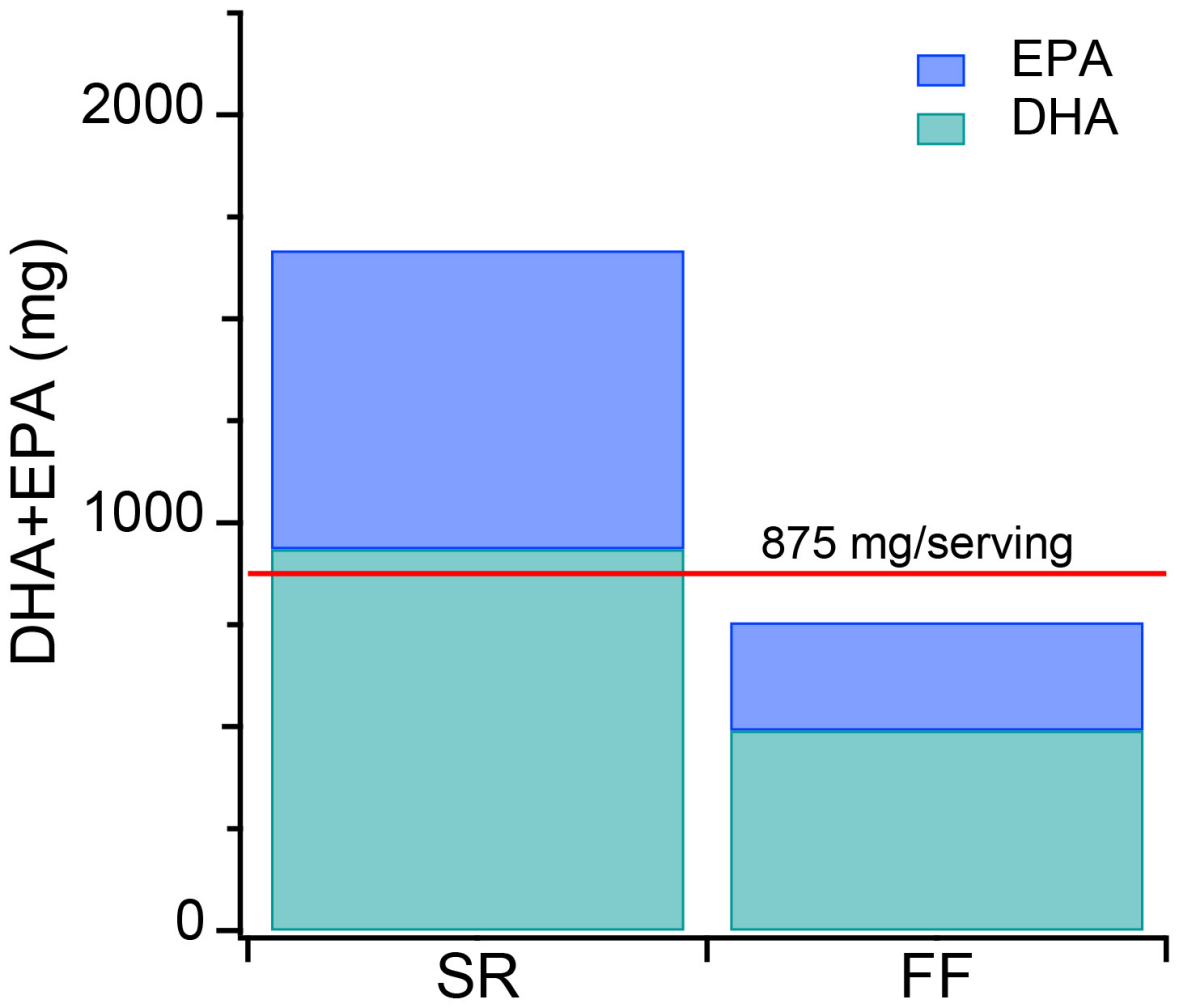
The estimated DHA+EPA content of a 3-oz. (85 g) serving of farmed Atlantic salmon using values from SR and FF. The red line indicates the per-serving value needed to meet dietary recommendations.

The findings of this study have immediate practical implications for dietary surveillance using FoodData Central (Figure 8). The dietary reference intake for DHA+EPA is 250 mg/day (48, 49), or 875 mg/serving assuming two servings of fish per week, the red line in Figure 8. Using the SR values for a 3 oz (85 g) serving of farmed salmon, that intake is easily met with 1,670 mg of DHA+EPA per serving, however using the FF values the intake misses the mark by 14%, with only 756 mg DHA+EPA per serving (Figure 8). With the caveat that the FF sample collection was not a complete nationally representative survey, the N3 fatty acid values for farmed Atlantic are different between SR Legacy and Foundation Foods. Therefore, a broader and more representative study is warranted to assess whether legacy SR values should be updated or replaced. This update would flow directly to FNDDS and, through it, to the dietary intake estimates derived from NHANES (2). Given that salmon is among the most consumed seafood species in the United States and is frequently recommended as a source of N3 fatty acids, the accuracy of these values has outsized relevance for dietary guidance (48, 50).

More broadly, this study demonstrates that food composition databases are not static repositories. The agricultural systems that produce the foods described in these databases are dynamic, and significant compositional changes can occur within a generation of data collection. The FF program's individual-sample approach provides the analytical resolution to detect some changes, while the framework presented here (a 2σ concordance criterion) offers a possible method for determining when such changes are consequential enough to warrant updating the database or engaging in a larger study with larger nationally representative sample collection.

## 5 Conclusions

This study establishes a practical framework for evaluating concordance between the USDA Foundation Foods and Standard Reference Legacy datasets, based on a two-standard-deviation criterion that distinguishes complementary data from data that signal genuine compositional change. Applied to fatty acid analysis of five commercially important fish species, the framework identifies farmed Atlantic salmon as a food whose composition appears to have changed sufficiently to render the legacy SR fatty acid values inaccurate. The decreases in EPA (63.8%), DPA (58.5%), and DHA (47.5%), concurrent with increases in plant-derived fatty acids consistent with changes in aquaculture feed practices, have direct implications for dietary intake estimates and nutritional guidance. For wild sockeye salmon, farmed channel catfish, tilapia, and cod, the SR and FF datasets are largely complementary, with FF providing valuable information about biological variability that the legacy composite sampling design could not capture. These findings underscore the necessity of continuous investment in food composition analysis to ensure that the data underpinning national dietary surveillance.

## Data Availability

The datasets presented in this study can be found in online repositories. The names of the repository/repositories and accession number(s) can be found in the article/supplementary material.
