# Supplementary material for: Foundation Foods reveals significant changes in farmed Atlantic salmon relative to Standard Reference Legacy data
Source: Front Nutr. 2026 Aug 3;13:1870100. doi: 10.3389/fnut.2026.1870100 (PMC13478241; doi:10.3389/fnut.2026.1870100)
Supplement: Supplementary file 1 [file Table_2.docx]

**Supplemental Data**

Foundation Foods reveals significant changes in farmed Atlantic salmon relative to Standard Reference legacy data.

Michael R. Bukowski*, Robert Goldschmidt, W. Craig Byrdwell, Kyle McKillop, Katherine M. Phillips, Pamela Pehrsson, Patrick Sullivan, Naomi Fukagawa

USDA-Agricultural Research Service, Beltsville Human Nutrition Center,

Beltsville, MD;

Department of Biochemistry, Virginia Tech, Blacksburg, VA

Corresponding Author:

Michael R. Bukowski, Ph.D.

Methods and Application of Food Composition Laboratory

Beltsville Human Nutrition Research Center

USDA-ARS-NEA, 10300 Baltimore Ave., Building 307C

Beltsville, MD 20705

Tel:(301) 504-5720

Fax: (301)-504-8314

Email: [michael.bukowski@usda.gov](mailto:michael.bukowski@usda.gov)

Supplemental Table 1. Fatty acid species with level of identification, asterisks indicate species reported in Foundation Foods.

| **Saturated Fatty Acid** | **Full Structural** | FA 4:0 (butyric)* |  | **Polyunsaturated Fatty Acid** | **Full Structural** | FA 18:2 (9Z,12Z) (linoleic)* |
| --- | --- | --- | --- | --- | --- | --- |
|  |  | FA 5:0 |  |  |  | FA 18:3 (6Z,9Z,12Z) (γ-linolenic)* |
|  |  | FA 6:0 (caproic)* |  |  |  | FA 18:3 (9Z,12Z,15Z) (α-linolenic)* |
|  |  | FA 7:0 |  |  |  | FA 20:2 (11Z,14Z) |
|  |  | FA 8:0 (caprylic)* |  |  |  | FA 20:3 (8Z,11Z,14Z) |
|  |  | FA 9:0 |  |  |  | FA 20:3(11Z,14Z,17Z) |
|  |  | FA 10:0 (capric)* |  |  |  | FA 20:4 (5Z,8Z,11Z,14Z) |
|  |  | FA 11:0 |  |  |  | FA 20:5 (5Z,8Z,11Z,14Z,17Z) (eicosapentaenoic)* |
|  |  | FA 12:0 (lauric)* |  |  |  | FA 22:2 (13Z,16Z) |
|  |  | FA 13:0 |  |  |  | FA 22:3(13Z, 16Z, 19Z) |
|  |  | FA 14:0 (myristic)* |  |  |  | FA 22:5 (7Z,10Z,13Z,16Z,19Z) (docosapentaeneoic)* |
|  |  | FA 15:0 |  |  |  | FA 22:6 (3Z,6Z,9Z,12Z,15Z,18Z) (docosahexaeneoic) * |
|  |  | FA 16:0(palmitic)* |  |  | **Species Only** | FA 16:2(Isomer1) |
|  |  | FA 17:0 |  |  |  | FA 16:2(Isomer2) |
|  |  | FA 18:0 (stearic)* |  |  |  | FA 16:2(Isomer3) |
|  |  | FA 19:0 |  |  |  | FA 16:2(Isomer 4) |
|  |  | FA 20:0* |  |  |  | FA 16:3(Isomer 1) |
|  |  | FA 22:0* |  |  |  | FA 18:2 (isomer 1) |
|  |  | FA 23:0 |  |  |  | FA 18:2 (isomer 2) |
|  |  | FA 24:0* |  |  |  | FA 18:2 (Z,Z isomer 1) |
|  |  | FA 25:0 |  |  |  | FA 18:2 (isomer 3) |
|  |  | FA 26:0 |  |  |  | FA 18:2 FA LA |
| **Monounsaturated Fatty Acid** | **Full Structural** | FA 11:1 |  |  |  | FA 18:3 (Z,Z,E isomer) |
|  |  | FA 12:1 (11Z) |  |  |  | FA 18:3 (isomer 1) |
|  |  | FA 13:1 |  |  |  | FA 18:4 (Isomer 1) |
|  |  | FA 14:1 (9Z) |  |  |  | FA 18:4 (Isomer 3) |
|  |  | FA 15:1 (10Z) |  |  |  | FA 18:4 (Isomer 3) |
|  |  | FA 16:1 (8Z) |  |  |  | FA 20:2 (Isomer1) |
|  |  | FA 16:1 (9Z)* |  |  |  | FA 20:2 (Isomer2) |
|  |  | FA 16:1 (10Z) |  |  |  | FA 20:2 (Isomer3) |
|  |  | FA 16:1 (11Z)* |  |  |  | FA 20:2 (Isomer4) |
|  |  | FA 16:1 (12Z) |  |  |  | FA 20:3 (Isomer1) |
|  |  | FA 16:1 (13Z) |  |  |  | FA 20:3 (Isomer2) |
|  |  | FA 17:1 (10Z) |  |  |  | FA 20:4(Isomer 1) |
|  |  | FA 18:1(10Z) |  |  |  | FA 22:3(Isomer 1) |
|  |  | FA 18:1(9Z) (oleic)* |  |  |  | FA 22:3(Isomer 2) |
|  |  | FA 18:1(11Z)* |  |  |  | FA 22:3(Isomer 3) |
|  |  | FA 18:1(12Z) |  |  |  | FA 22:4(Isomer) |
|  |  | FA 18:1(13Z) |  |  |  | FA 22:4(Isomer 1) |
|  |  | FA 18:1(14Z) |  |  |  | FA 22:5(Isomer 1) |

Supplemental Table 1. (continued) Fatty acid species with level of identification.

| **Monounsaturated Fatty Acid** | **Full Structural** | FA 18:1 (15Z) |  |  |  | FA 22:5(Isomer 2) |
| --- | --- | --- | --- | --- | --- | --- |
|  |  | FA 19:1 (7Z) |  | **E-Monounsaturated Fatty Acid** | **Full Structural** | FA 16:1 (13E) |
|  |  | FA 20:1(5Z)* |  |  |  | FA 16:1 (4E) |
|  |  | FA 20:1(8Z)* |  |  |  | FA 16:1 (5E) |
|  |  | FA 20:1(9Z) * |  |  |  | FA 16:1 (7E) and (6E) |
|  |  | FA 20:1(10Z)* |  |  |  | FA 16:1 (8E) |
|  |  | FA 20:1(11Z)* |  |  |  | FA 16:1 (9E) |
|  |  | FA 22:1(13Z) |  |  |  | FA 16:1 (10E) |
|  |  | FA 24:1 (15Z) |  |  |  | FA 16:1 (11E) |
|  | **Species Only** | FA 14:1 (isomer 1) |  |  |  | FA 16:1(14E) |
|  |  | FA 14:1 (isomer 2) |  |  |  | FA 18:1 (4E) |
|  |  | FA 14:1 (isomer 3) |  |  |  | FA 18:1 (5E) |
|  |  | FA 14:1 (isomer 4) |  |  |  | FA 18:1 (6E) and (8E) |
|  |  | FA 15:1 (isomer 1) |  |  |  | FA 18:1(9E) |
|  |  | FA 15:1 (isomer 2) |  |  |  | FA 18:1(10E) |
|  |  | FA 16:1 (isomers) |  |  |  | FA 18:1(11E) |
|  |  | FA 17:1(Isomer1) |  |  |  | FA 18:1(12E) |
|  |  | FA 17:1(Isomer2) |  |  |  | FA 18:1 (15E) |
|  |  | FA 17:1(Isomer3) |  |  |  | FA 18:1 (16E) |
|  |  | FA 17:1(Isomer4) |  | **E-Polyunsaturated Fatty Acid** | **Full Structural** | FA 18:2 (9E,12E) |
|  |  | FA 17:1(Isomer5) |  |  |  | FA 18:2 (9Z,12E) |
|  |  | FA 17:1(Isomer6) |  |  |  | FA 18:2 (9E,12Z) |
|  |  | FA 17:1(Isomer7) |  |  |  | FA 18:2 (11E,15Z) |
|  |  | FA 17:1(Isomer8) |  |  | **Species Only** | FA 18:2 (E,E isomer 1) |
|  |  | FA 17:1(Isomer9) |  |  |  | FA 18:2 (E,E isomer 2) |
|  |  | FA 19:1 (isomer 1) |  |  |  | FA 18:2 (E,E isomer 3) |
|  |  | FA 19:1 (isomer 2) |  |  |  | FA 18:2 (E,E isomer 4) |
|  |  | FA 19:1 (isomer 3) |  | **Branched Chain Fatty Acid** | **Full Structural** | FA 12:0,11-Me |
|  |  | FA 19:1 (isomer 4) |  |  |  | FA 12:0,10-Me |
|  |  | FA 19:1 (isomer 5) |  |  |  | FA 13:0,12-Me |
|  |  | FA 19:1 (isomer 6) |  |  |  | FA 14:0, 13-Me |
|  |  | FA 19:1 (isomer 7) |  |  |  | FA 14:0, 12-Me |
|  |  | FA 20:1 (Isomer2) |  |  |  | FA 15:0,14-Me |
|  |  | FA 20:1 (Isomer3) |  |  |  | FA 16:0,15-Me |
|  |  | FA 20:1 (Isomer4) |  |  |  | FA 16:0,14-Me |
|  |  | FA 22:1(putative) |  |  |  | FA 17:0(16-Me) |
|  |  | FA 22:1(Isomer 1) |  |  | **Species Only** | FA 17:0 (Isomer 1) |
|  |  | FA 22:1(Isomer 2) |  |  |  | FA 17:0 (Isomer 2) |
|  |  | FA 22:1(Isomer 3) |  |  |  | FA 17:0 (Isomer 3) |
|  |  | FA 24:1 (Isomer 1) |  |  |  | FA 17:0 (Isomer 4) |
|  |  | FA 24:1 (Isomer 2) |  |  |  | FA 18:0(Isomer1) |
|  |  |  |  | **Other** | **Species Only** | Pristanic acid (Isomer 1) |
|  |  |  |  |  |  | Pristanic acid (Isomer 2) |
|  |  |  |  |  |  | Phytanic acid (Isomer 1)  Phytanic acid (Isomer 2) |

Supplementary Table 2 (in Excel file). Metadata and measured fatty acid species for samples gathered for Foundation Foods

Supplementary Table 3. Mean, SD, range, and 95% confidence intervals for nutritionally relevant fatty acids in farmed Atlantic salmon Foundation Foods samples. Quantities are expressed in grams/100 grams uncooked fillet.

| Sample |  | FA 18:1(9Z) | FA 18:2 | FA 18:3 | FA 20:5 | FA 22:5 | FA 22:6 | EPA + DHA |
| --- | --- | --- | --- | --- | --- | --- | --- | --- |
|  |  | oleic acid | linoleic acid (N6) | α-linolenic acid (N3) | EPA | DPA | DHA |  |
|  |  |  |  |  | (N3) | (N3) | (N3) | (N3) |
| Atlantic salmon, farmed | Mean | 4.26 | 2.152 | 0.510 | 0.312 | 0.163 | 0.577 | 0.889 |
|  | St. Dev. | 0.41 | 0.351 | 0.083 | 0.056 | 0.026 | 0.058 | 0.108 |
|  | 95% CI | 3.93-4.50 | 1.91-2.40 | 0.452-0.567 | 0.274-0.351 | 0.144-0.181 | 0.537-0.617 | 0.814-0.964 |
|  | Range | 3.61-4.75 | 1.67-2.75 | 0.379-0.626 | 0.228-0.420 | 0.116-0.211 | 0.524-0.699 | 0.752-1.112 |
| Sockeye Salmon, wild | Mean | 0.582 | 0.049 | 0.020 | 0.210 | 0.068 | 0.324 | 0.534 |
|  | St. Dev. | 0.072 | 0.008 | 0.005 | 0.065 | 0.020 | 0.087 | 0.145 |
|  | 95% CI | 0.532-0.632 | 0.044-0.055 | 0.017-0.024 | 0.165-0.255 | 0.054-0.082 | 0.264-0.385 | 0.434-0.635 |
|  | Range | 0.491-0.693 | 0.041-0.062 | 0.013-0.027 | 0.081-0.286 | 0.026-0.093 | 0.214-0.480 | 0.296-0.766 |
| Tilapia, farmed | Mean | 0.607 | 0.248 | 0.018 | 0.002 | 0.007 | 0.029 | 0.032 |
|  | St. Dev. | 0.267 | 0.073 | 0.006 | 0.0008 | 0.002 | 0.007 | 0.007 |
|  | 95% CI | 0.422-0.792 | 0.192-0.293 | 0.014-0.022 | 0.001-0.002 | 0.006-0.009 | 0.025-0.034 | 0.027-0.037 |
|  | Range | 0.318-0.954 | 0.119-0.374 | 0.006-0.023 | 0.001-0.003 | 0.006-0.011 | 0.017-0.038 | 0.018-0.039 |
| Atlantic cod, wild | Mean | 0.011 | 0.00097 | 0.00023 | 0.0181 | 0.0026 | 0.0533 | 0.071 |
|  | St. Dev. | 0.004 | 0.00058 | 0.00021 | 0.0075 | 0.0005 | 0.0204 | 0.028 |
|  | 95% CI | 0.009-0.014 | 0.00054-0.00141 | 0.00008-0.00039 | 0.0126-0.0236 | 0.0022-0.0030 | 0.0381-0.0684 | 0.021-0.092 |
|  | Range | 0.006-0.016 | 0.00037-0.00171 | 0.00005-0.00064 | 0.0072-0.0300 | 0.0014-0.0034 | 0.0269-0.0849 | 0.034-0.115 |
| Channel catfish, farmed | Mean | 3.02 | 0.938 | 0.062 | 0.0138 | 0.0133 | 0.0447 | 0.058 |
|  | St. Dev. | 0.63 | 0.175 | 0.010 | 0.0101 | 0.0050 | 0.0184 | 0.027 |
|  | 95% CI | 2.58-3.46 | 0.817-1.059 | 0.055-0.069 | 0.0076-0.020 | 0.010-0.017 | 0.032-0.057 | 0.040-0.077 |
|  | Range | 2.31-4.46 | 0.699-1.288 | 0.051-0.085 | 0.0064-0.0345 | 0.0089-0.024 | 0.027-0.086 | 0.033-0.121 |
